# Laser-Assisted Gingival Depigmentation: A Case Report

**DOI:** 10.7759/cureus.51670

**Published:** 2024-01-04

**Authors:** Shrishti S Salian, Prasad V Dhadse, Ruchita T Patil

**Affiliations:** 1 Periodontics and Implantology, Sharad Pawar Dental College, Datta Meghe Institute of Higher Education and Research, Wardha, IND

**Keywords:** melanin, aesthetics, hyperpigmentation, diode laser, gingival depigmentation

## Abstract

The gingiva's colour varies in different individuals and is assumed to be related to cutaneous pigmentation. The most frequent natural pigment causing endogenous gingival pigmentation is melanin, a brown pigment. Depigmentation is a therapy of choice when individuals are concerned about their appearance and demand it for their aesthetic satisfaction. It is not a clinical indication. This article demonstrates gingival depigmentation using a laser diode with a 90-day follow‑up.

A 23-year-old male patient visited the Periodontology Department, complaining of poor aesthetics owing to dark-coloured gums. Depigmentation with a laser diode was selected as the treatment plan for both the maxillary and mandibular arches, at an interval of a week.

The choice of a procedure is largely influenced by the gingival thickness, the clinician's experience, the patient's preferences, and the rate of recurrence. According to reports, using lasers produces better aesthetic outcomes and has a low recurrence rate.

## Introduction

The health and aesthetics of gingiva are crucial aspects of a smile, along with lips, teeth, and face [1]. The shading of the gingiva differs from person to person and is thought to be correlated with cutaneous pigmentation. Similar to the manner in which the texture and colour of skin vary among different ethnicities and geographical areas, it ranges from light to dark brown or black. In all ethnicities, gingival melanin pigmentation is present [2].

The primary determinants of gingival colour are the thickness of the gingival epithelium, the amount and size of vasculature, the level of keratinization, and the pigments that are present. The most frequent natural pigment causing endogenous gingival pigmentation is melanin, a brown pigment. The gingiva is also the mucosa's most often pigmented area. Melanin colouring arises in the basal layer of the gingival epithelium due to the entanglement of melanin granules generated by melanoblasts between epithelial cells. [3].

Gingival hyperpigmentation is also known as physiological or racial gingival pigmentation as it is thought to be a genetic feature in some groups, regardless of age or gender. Furthermore, dark-skinned ethnic groups frequently encounter gingival melanosis (neurofibromatosis), and it is frequently seen in diseases including Addison's syndrome, Peutz syndrome, and Jegher's and Von Recklinghausen's disease [4].

Gingival depigmentation is a periodontal plastic surgical procedure through which the gingival hyperpigmentation is removed or reduced by various techniques. Depigmentation is a therapy of choice when individuals are concerned about their appearance and want it. It is not a clinical indication [5]. Several depigmentation methods have been used, such as chemical methods like using agents like alcohols, phenols, and ascorbic acid, conventional methods like surgical scalpel technique, gingival abrasion, free gingival grafting, acellular dermal matrix allograft, laser depigmentation, electrosurgery, cryosurgery, and radiosurgery [5-7]. This article demonstrates gingival depigmentation using a laser diode with 90 days follow‑up.

## Case presentation

**Figure 1:**
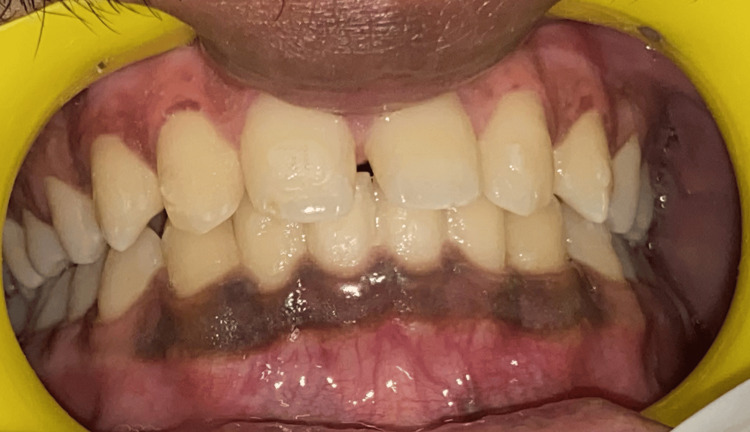
Hyperpigmented gingiva in the mandibular arch

**Figure 2:**
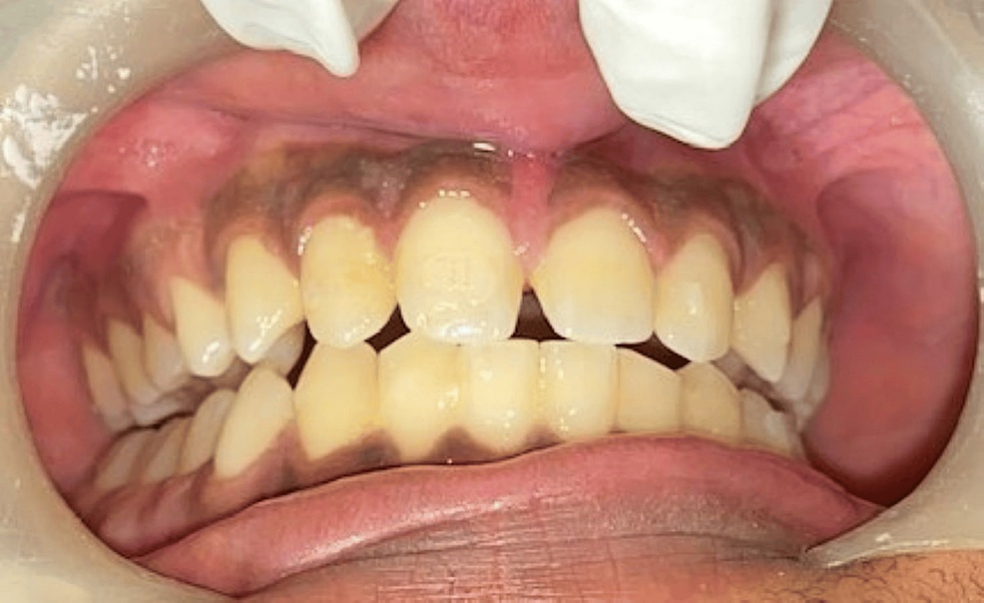
Hyperpigmented gingiva in the maxillary arch

A male patient, 23 years old, reported to the Department of Periodontics and Implantology, complaining of poor aesthetics owing to dark gums (Figures 1, 2). Additional historical accounts indicated that it had existed since childhood, indicating physiological melanin pigmentation. Overall, the patient’s oral hygiene was systemically healthy and good.

**Figure 3:**
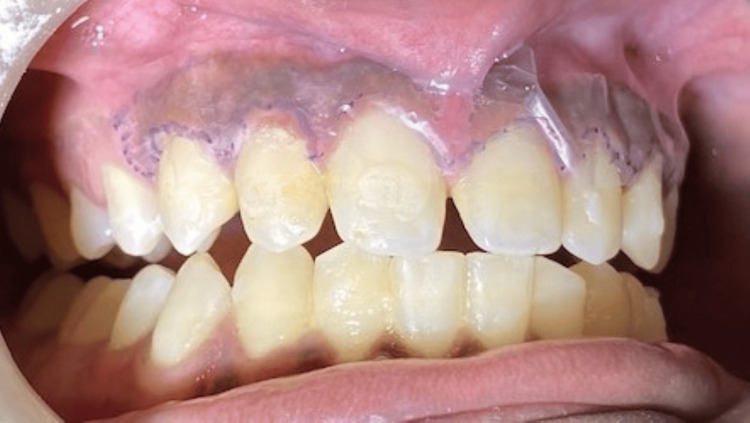
Stent prepared for the maxillary arch

**Figure 4:**
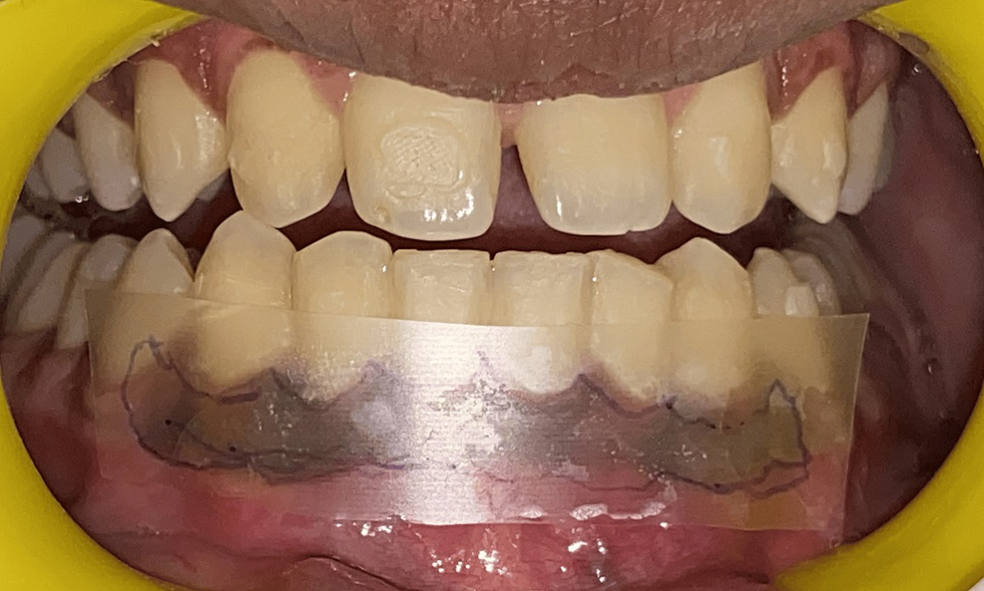
Stent prepared for the mandibular arch

**Figure 5:**
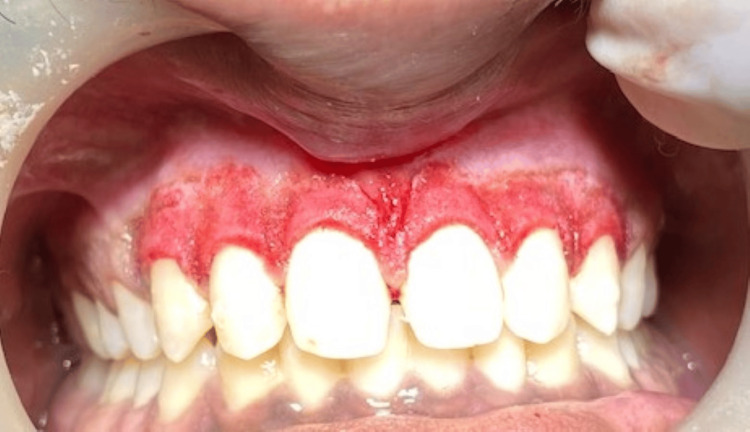
Depigmentation using a laser diode in the maxillary arch

**Figure 6:**
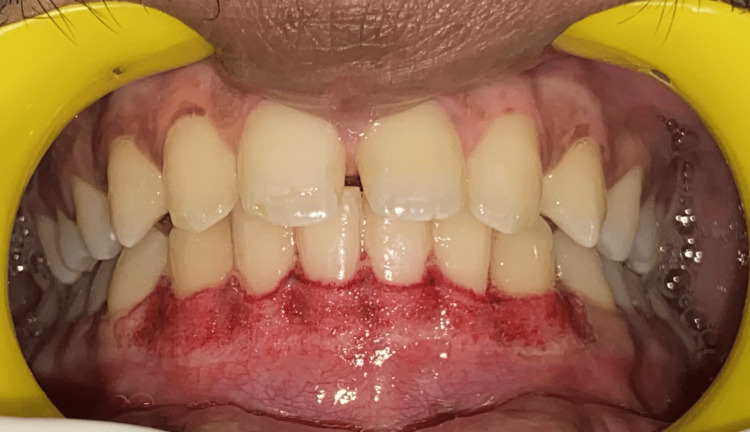
Depigmentation using a laser diode in the mandibular arch

**Figure 7:**
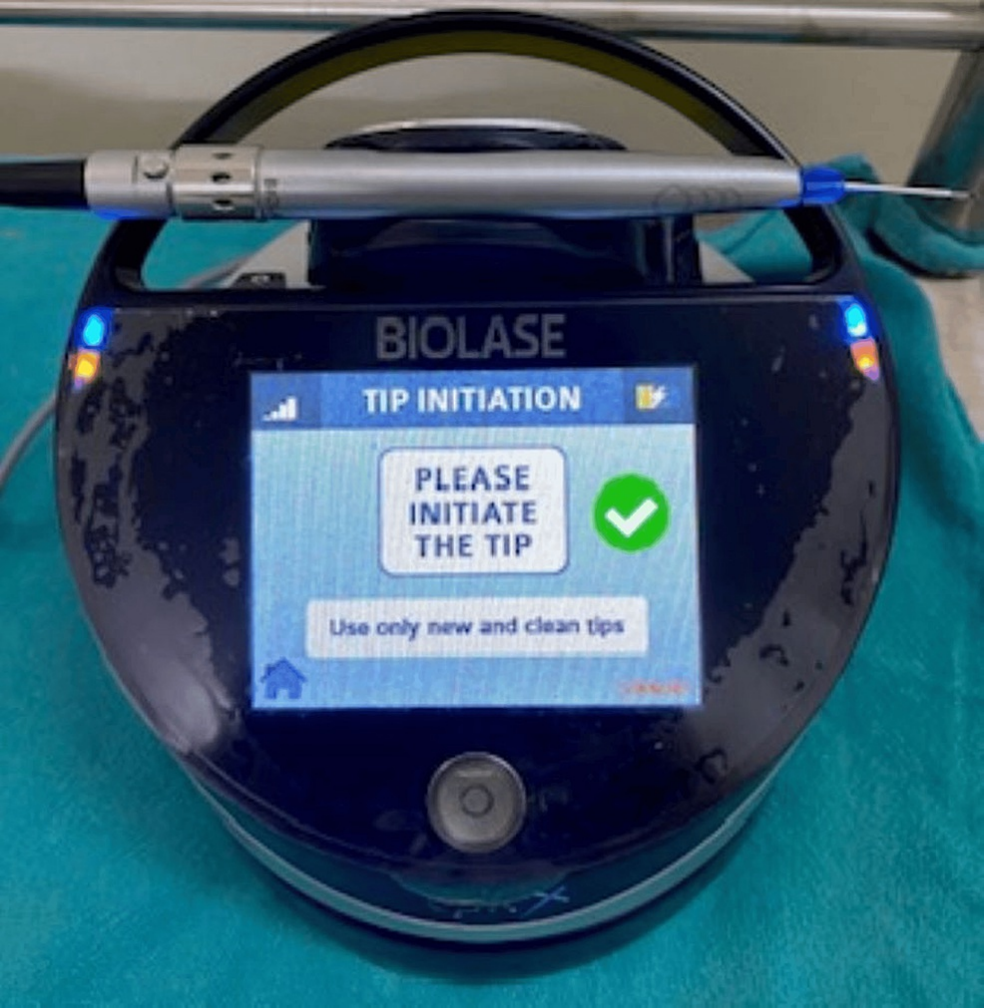
The laser diode used in the procedure Biolase, Lake Forest, California, United States

In phase one therapy, scaling was done and out of the treatment options given, the patient opted for laser depigmentation. Local infiltration was administered. A stent was made (Figures 3, 4) and a laser diode was used for depigmentation, extending from the right canine to the left canine in both the maxillary and mandibular arches (Figures 5, 6). The maxillary arch was treated first, followed by the mandibular arch seven days later. The laser used is shown in Figure 7. In order to avoid overheating, depigmentation was carried out horizontally, with the laser tip in contact mode on the pigmented gingiva and parallel to the root surfaces. The depigmented area was then washed with gauze soaked in saline. 

**Figure 8:**
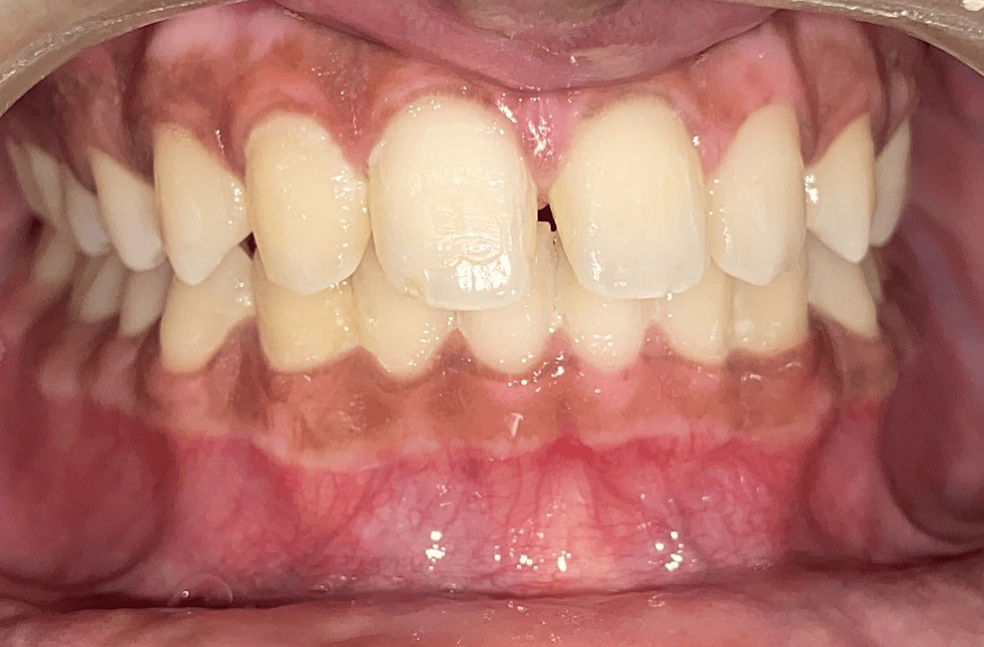
Three-month post-operative clinical view

**Figure 9:**
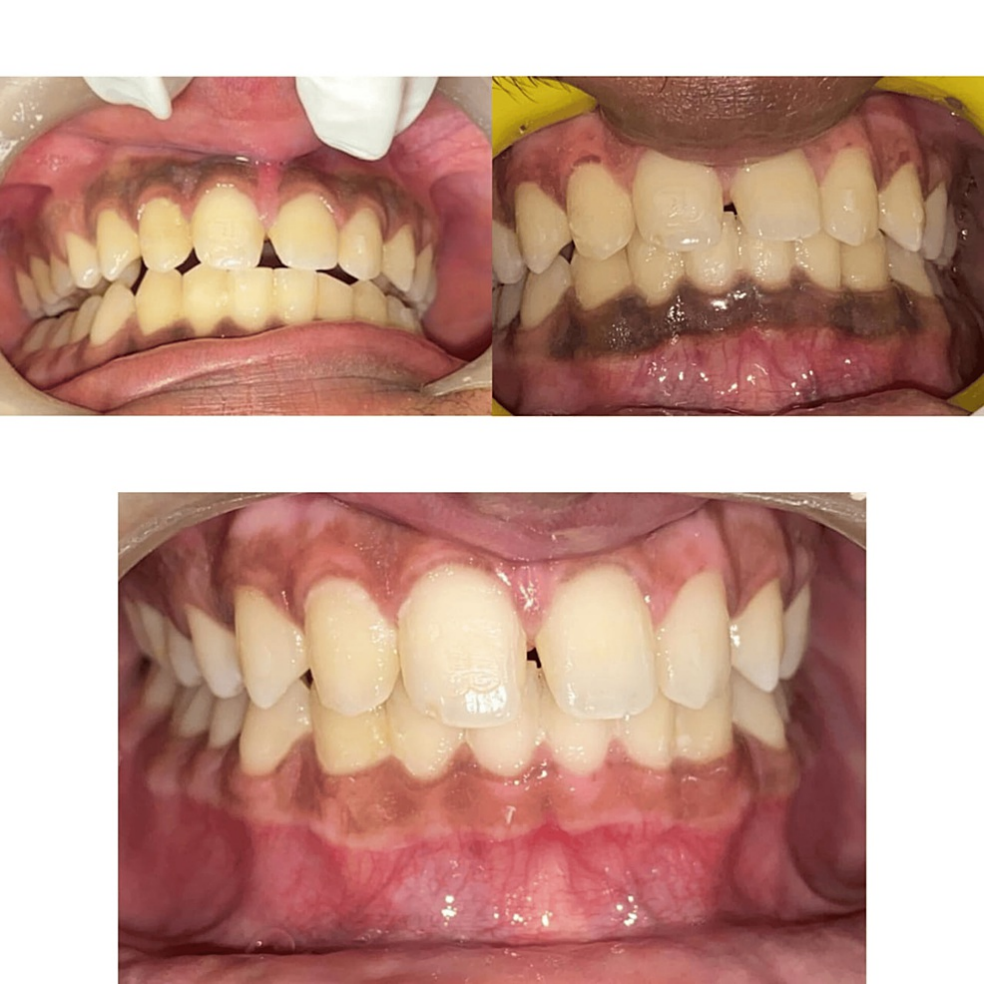
Comparative pre- and post-case photos

The patient felt at ease throughout the surgery, and there was no bleeding. A periodontal pack was placed and postoperative instructions were given to the patient. The patient was recalled after one week for re‑evaluation, wherein, the patient reported no pain in the area of interest. The periodontal pack was removed. Uncomplicated healing ensued in both arches. On a three‑month postoperative follow‑up, it seemed normal since the gingiva was firm, pink, and healthy (Figure 8). The patient was very satisfied with the results and no recurrence was reported. Figure 9 shows the comparative pre- and post-case photos.

## Discussion

Although there is a lot of interest in employing lasers for gingival depigmentation, there is not much information available about their use. One of the efficient strategies for treating gingival hyperpigmentation is laser therapy. Carbon dioxide (CO2, 10,600 nm), neodymium: yttrium-aluminium-garnet (Nd: YAG), and diode (980 nm) lasers are the most often utilised lasers for gingival depigmentation. When it comes to treating gingival pigmentation, the ablative method involves surgically removing the gingival epithelium, unlike the non-ablative laser treatment that relies on the concept of selective photothermolysis using some wavelengths of the near-infrared that melanin-like diodes strongly absorb [3].

The thermal impact on soft tissue can be influenced by the emission parameters of individual laser systems, even though the absorption rate is determined by each laser's wavelength, thermal outcome, and tissue characteristics. Even if melanin and haemoglobin absorb most of the 980 nm diode laser, photothermal soft tissue ablation with a diode of that wavelength is difficult to do therapeutically short of causing significant collateral thermal damage because of the high energy density level needed. As a result, in order to boost the tip's temperature, the diode laser tip has to be started using low power settings (0.8 W). When the laser point comes into contact with the desired tissue, this provides an opportunity to use the thermal conducting effect to achieve tissue excision [7].

During the first week, we saw a rapid and full re-epithelialization. This could be explained by the fact that diode lasers cause less heat damage to gingival tissue. According to Kaya et al., the time needed for treatment by the diode laser is less compared to that by the erbium-doped yttrium-aluminium-garnet (Er: YAG) laser. While Kaya et al. required several weekly laser therapy sessions to eliminate the extra pigmentation, the depigmentation process was finished in a single session in the current case [8].

In general, results after one month were reported by patients with high levels of satisfaction with good esthetics. Low recurrence rates and easy access make it a safe and efficient treatment option. Less post-operative problems, such as discomfort or pain, haemorrhage, oedema, infection as well as slowed wound healing, are demonstrated by lasers, along with improved haemostatic activity and good vision at the surgical site. The only disadvantage may be the high cost of the lasers. [9,10].

Diode lasers can penetrate deeper and are found in the melanin absorption spectrum. The diode laser's deep thermal impact causes unintentional irradiation that seals blood vessels in the surrounding tissue and may cause melanocyte migration to be delayed. Moreover, the pigment-containing cells, such as melanophages or melanophores, which may have gotten entrapped in the lamina propria may absorb the diode laser. It also has particular impacts on the cells that decrease their activity [11]. 

A comparison of the effectiveness of laser and scalpel methods for treating gingival hyperpigmentation was presented by Hassan et al. (2022). The right side underwent scalpel treatment while the left side of a female patient, age 23, underwent diode laser treatment. The authors came to the conclusion that both strategies had comparable healing outcomes and that there was no recurrence with any of the therapies they looked at. While the charred layer functioned as a dressing to control the bleeding, the scalpel procedure left a bleeding region that needed to be inspected post-surgery [12].

Two female patients had depigmentation treatment using an 810 nm diode laser in a case series by Elemek E. At weeks one, four, and 12, the patients were called back to assess the rate of healing and recurrence. According to this study, depigmentation using an 810 nm diode laser is effective in terms of patient comfort and aesthetics [13]. 

## Conclusions

The majority of patients who need depigmentation therapy have excessive gingival display. Depending on the procedure used and the follow-up period, relapse or repigmentation is a serious risk. The choice of a procedure is largely influenced by the clinician's experience, the preferences of patients, and the rate of recurrence. According to reports, using lasers produces better aesthetic outcomes and has a low recurrence rate, but more studies regarding effectiveness and efficiency of available techniques are required.
